# Supplementary material for: Complex‐centric proteome profiling by SEC‐SWATH‐MS
Source: Mol Syst Biol. 2019 Jan 14;15(1):e8438. doi: 10.15252/msb.20188438 (PMC6346213; doi:10.15252/msb.20188438)
Supplement: Supplementary file 8 — Dataset EV7 [file MSB-15-e8438-s008.zip › feature_plots_string/Q8WVM8.pdf]

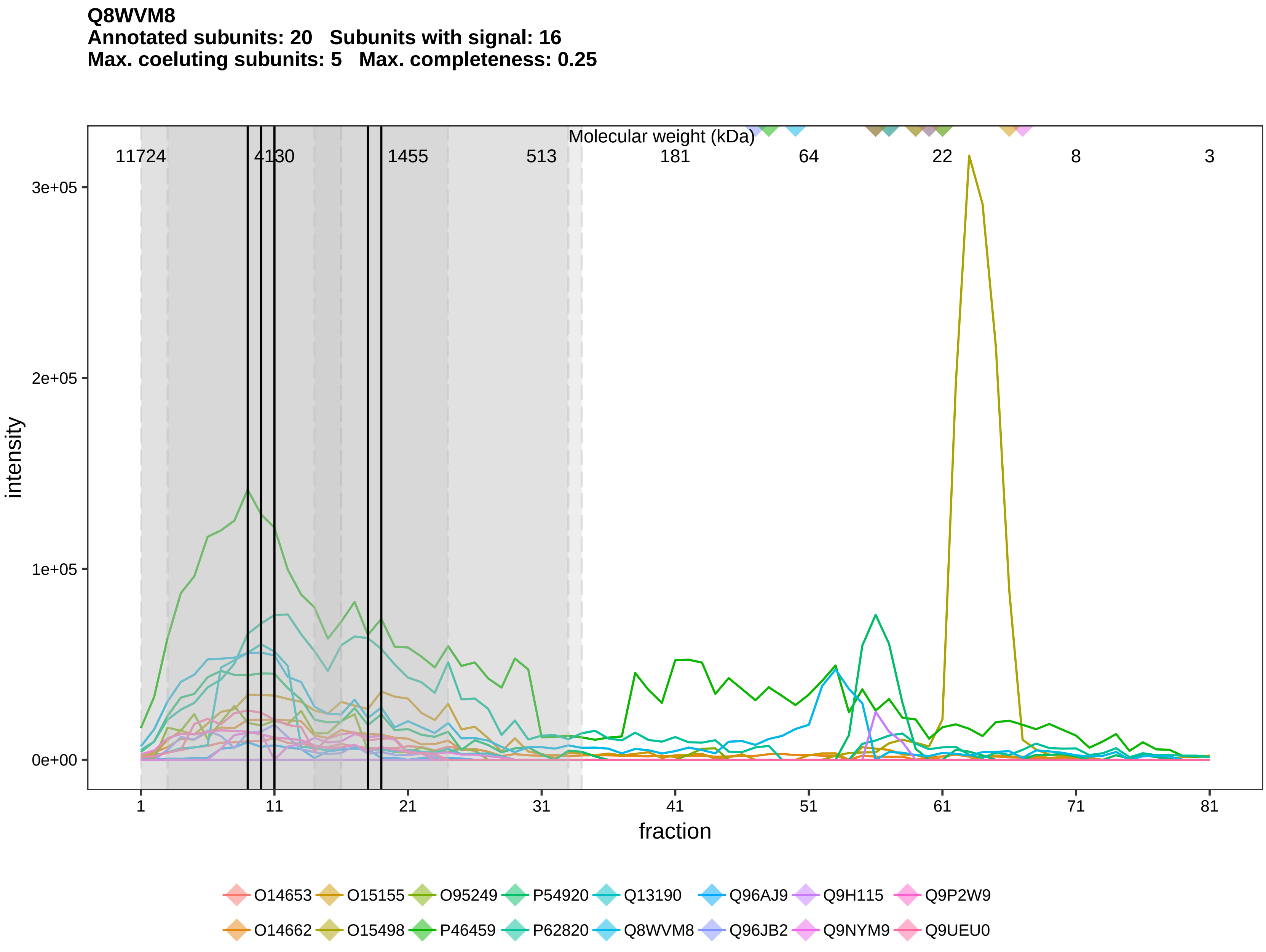
Q8WVM8
Annotated subunits: 20 Subunits with signal: 16
Max. coeluting subunits: 5 Max. completeness: 0.25
Molecular weight (kDa)
11724
4130
1455
513
181
64
22
8
3
intensity
3e+05
2e+05
1e+05
0e+00
fraction
1
11
21
31
41
51
61
71
81
O14653
O15155
O95249
P54920
Q13190
Q96AJ9
Q9H115
Q9P2W9
O14662
O15498
P46459
P62820
Q8WVM8
Q96JB2
Q9NYM9
Q9UEU0
